# Functional analysis of the *SlERF01* gene in disease resistance to *S. lycopersici*

**DOI:** 10.1186/s12870-020-02588-w

**Published:** 2020-08-15

**Authors:** Huanhuan Yang, Fengyi Shen, Hexuan Wang, Tingting Zhao, He Zhang, Jingbin Jiang, Xiangyang Xu, Jingfu Li

**Affiliations:** College of Horticulture and Landscape Architecture, Northeast Agricultural University, Mucai Street 59, Xiangfang District, Harbin, 150030 China

**Keywords:** Tomato, *SlERF01*, Resistance response, *S. Lycopersici*

## Abstract

**Background:**

Tomato gray leaf spot caused by *Stemphylium lycopersici* (*S. lycopersici*) is a serious disease that can severely hinder tomato production. To date, only *Sm* has been reported to provide resistance against this disease, and the molecular mechanism underlying resistance to this disease in tomato remains unclear. To better understand the mechanism of tomato resistance to *S. lycopersici*, real-time quantitative reverse transcription-polymerase chain reaction (qRT-PCR)-based analysis, physiological indexes, microscopy observations and transgenic technology were used in this study.

**Results:**

Our results showed that the expression of *SlERF01* was strongly induced by *S. lycopersici* and by exogenous applications of the hormones salicylic acid (SA) and jasmonic acid (JA). Furthermore, overexpression of *SlERF01* enhanced the hypersensitive response (HR) to *S. lycopersici* and elevated the expression of defense genes in tomato. Furthermore, the accumulation of lignin, callose and hydrogen peroxide (H_2_O_2_) increased in the transgenic lines after inoculation with *S. lycopersici.* Taken together, our results showed that *SlERF01* played an indispensable role in multiple SA, JA and reactive oxygen species (ROS) signaling pathways to provide resistance to *S. lycopersici* invasion. Our findings also indicated that *SlERF01* could activate the expression of the *PR1* gene and enhance resistance to *S. lycopersici*.

**Conclusions:**

We identified the *SlERF01* gene, which encodes a novel tomato AP2/ERF transcription factor (TF). Functional analysis revealed that *SlERF01* positively regulates tomato resistance to *S. lycopersici*. Our findings indicate that *SlERF01* plays a key role in multiple SA, JA and ROS signaling pathways to provide resistance to invasion by *S. lycopersici.* The findings of this study not only help to better understand the mechanisms of response to pathogens but also enable targeted breeding strategies for tomato resistance to *S. lycopersici*.

## Background

During the long-term competitive relationship between plants and pathogens, plants have developed a series of defense mechanisms to resist the threat of pathogens, including bacteria, viruses, fungi and insects [1, 2]. Two defense systems, PAMP-triggered immunity (PTI) and effector-triggered immunity (ETI), have been established to prevent pathogenic invasion [3]. Many early signaling components of PTI and ETI activate a series of downstream integrated defense responses to prevent further damage [4]. In fact, substantial overlap of defense responses occur between PTI and ETI [5].

The various defensive signaling responses include reactive oxygen species (ROS) bursts and callose and lignin accumulation and lead to localized cell and tissue death [6, 7], which is referred to as the hypersensitive response (HR), at the site of pathogenic invasion to limit pathogen growth [8–10]. Therefore, the HR is associated with resistance gene (R gene)-triggered resistance, leading to localized cell and tissue death with corresponding downstream defense responses [11–13]. As a chemically reactive molecule, hydrogen peroxide (H_2_O_2_) can induce the HR [14], which is associated with subsequent lignin and callose accumulation, limiting the growth of pathogens by strengthening cell walls.

If plant defense responses are induced at the site of infection, the systemic defense response is activated in other plant tissues to prevent further invasion by the pathogen. Systemic acquired resistance (SAR) is characterized by long-lasting, broad-spectrum effects [15]; these effects can be triggered by PTI- and ETI-mediated pathogen recognition and are related to the levels of salicylic acid (SA) in local cells and distant tissues. Previous studies have shown that the defense hormone SA plays an essential role in the SAR signaling pathway by inducing SAR-related gene expression via the regulatory protein *NPR1* and a transcriptional coactivator [16].

Gray leaf spot disease, which is caused by *Stemphylium lycopersici* and is destructive fungal disease of plant species such as pepper, cotton, spinach and eggplant, is considered a major factor limiting the yield and quality of cultivated tomato fruit worldwide [17]. However, effective methods to control this disease are unavailable. Hence, the development of resistant cultivars is the most efficient strategy to control the gray leaf spot. Only the incompletely dominant gene *Sm* provides strong resistance to *S. lycopersici* [18]. Identification of other disease R genes and further application of these genes are urgently needed. In addition, the mechanism underlying the resistance of tomato to *S. lycopersici* remains poorly understood. Therefore, identification of the molecular mechanism underlying the *Sm*-mediated resistance response to *S. lycopersici* and other R genes is urgently needed for the breeding of resistant tomato cultivars.

AP2/ERF-like transcription factors (TFs) have been shown to play an important role in disease resistance to various pathogens [19]. To date, a total of 137 ERF domain-containing proteins have been identified in the tomato genome, most of which are involved in the response to biotic and abiotic stress or in response to hormones; however, only a few of these proteins have been characterized [20]. Evidence has indicated that ERF proteins induce the expression of pathogenesis-related (*PR*) genes by interacting with GCC boxes in the response to pathogens [21]. In tomato, Pti4–6 and LeERF1 interact with GCC boxes and regulate the expression of *PR* genes [22]. In addition, ERF1 is transcriptionally regulated by pathogens, ethylene (ET), and jasmonic acid (JA) and is induced synergistically by ET and JA. It is known that the SA signal transduction pathway can act antagonistically with the ET/JA pathway. Interestingly, the expression of Pti4 and AtERF1 is induced by SA as well as by JA and ET [23, 24]. These findings indicate that Pti4, Pti5 and Pti6 indirectly regulate the SA response and that the expression of Pti4/5/6 in Arabidopsis enhances the expression level of the SA-regulated *PR1* and *PR2* genes [11].

In this study, in attempts to better understand the mechanism underlying resistance to *S. lycopersici* in tomato, a novel tomato AP2/ERF TF, *SlERF01*, was identified. Our data showed that *SlERF01* is directly or indirectly involved in the defense response to *S. lycopersici* in tomato via multiple signaling regulatory networks. This study not only revealed the preliminary function of *SlERF01* but also provides a new R gene resource for cultivating resistant tomato varieties.

## Results

### Cloning and phylogenetic analysis of *SlERF01*

**Fig. 1:**
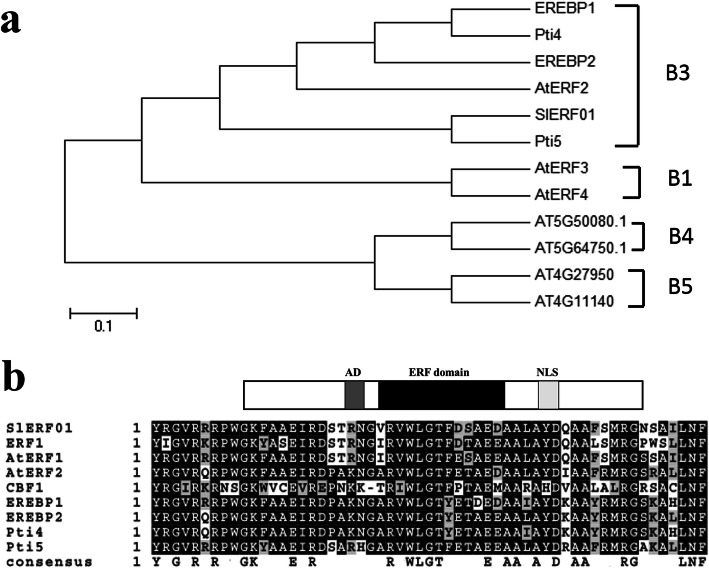
Phylogenetic tree and sequence alignment of *SlERF01***. a** Phylogenetic tree of *SlERF01* and other ERF proteins; the phylogenetic tree was constructed via ClustalW in conjunction with amino acid sequences of the AP2/ERF domain. Subfamilies of ERF proteins are divided by broken lines. The classification is described by Sakuma et al. (2002). **b** Alignment of *SlERF01* with other ERF proteins. *SlERF01* is composed of an ERF domain, a putative NLS and a putative AD, as shown in Fig. 1b. The black and light-gray colors represent identical and conserved amino acids, respectively, and the darker colors represent greater percentages of the same amino acid

The full-length CDS of *SlERF01* was cloned by PCR using cDNA derived from tomato (the PCR primers used are listed in Table S1). The CDS of *SlERF01* encodes a 240 amino acid protein that has one AP2/ERF domain and belongs to the ERF TF B-3 family (Fig. 1a). Analysis of the conserved protein sequence database revealed that only the ERF domain is conserved between *SlERF01* and other ERF proteins (Fig. 1b). Further analysis showed that *SlERF01* shares low similarity with other ERF proteins in terms of their whole putative protein sequences; however, sequence alignment revealed a high degree of homology in the ERF domain regions. Thus, the phylogenetic analysis results showed that *SlERF01* may encode a novel ERF protein that participates in the disease resistance response.

### Subcellular localization of *SlERF01*

**Fig. 2:**
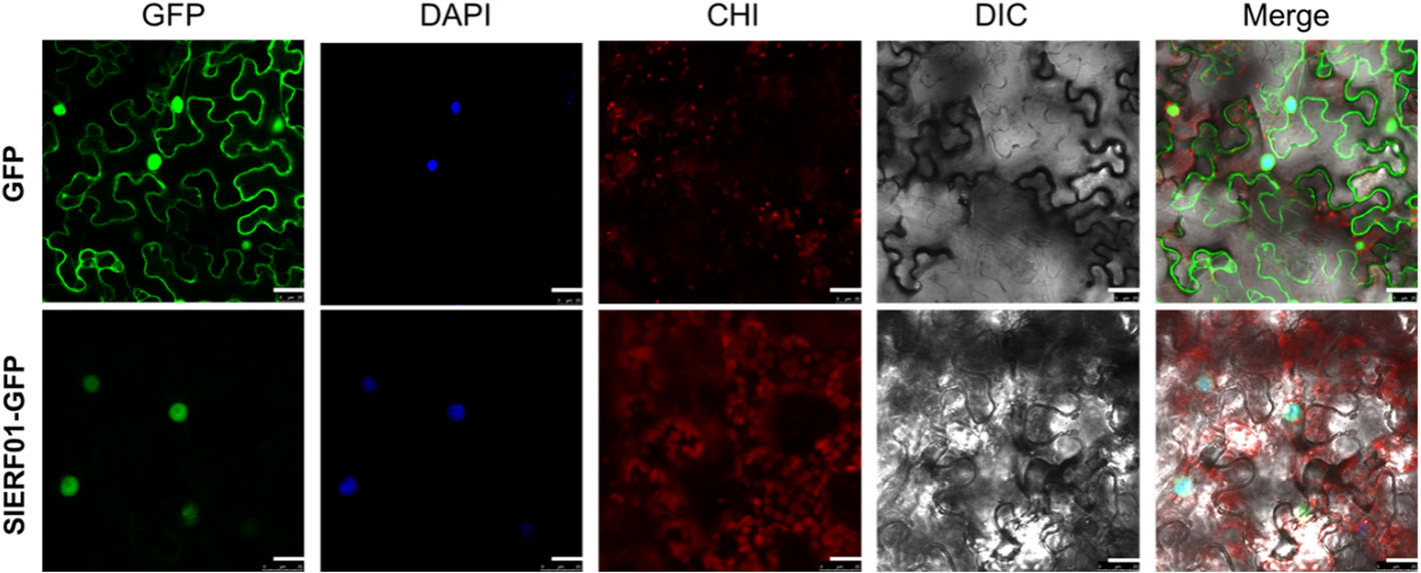
Subcellular localization of *SlERF01*. *SlERF01*-GFP was localized in the nucleus, and GFP was localized throughout the cells. GFP: green fluorescence field, DAPI: 4′,6-diamidino-2-phenylindole (DAPI) field (nuclear staining), CHI: chloroplast spontaneous fluorescence field, differential interference contrast (DIC): open field, Merge: superposition field. Light excitation wavelengths: GFP field: 488 nm, DAPI field: 358 nm, CHI field: 488 nm. The merged images were obtained 2 days after agroinfiltration. Bars = 25 μm

A *SlERF01****-***GFP fusion construct was developed. The *SlERF01*::GFP fusion construct was subsequently transformed into the *A. tumefaciens* GV3101 strain, with an empty GFP vector serving as a negative control. *N. benthamiana* leaves were then infected. The results showed that *SlERF01* localized to the nucleus (Fig. 2).

### *SlERF01* improves disease resistance against *S. lycopersici* in tomato

**Fig. 3:**
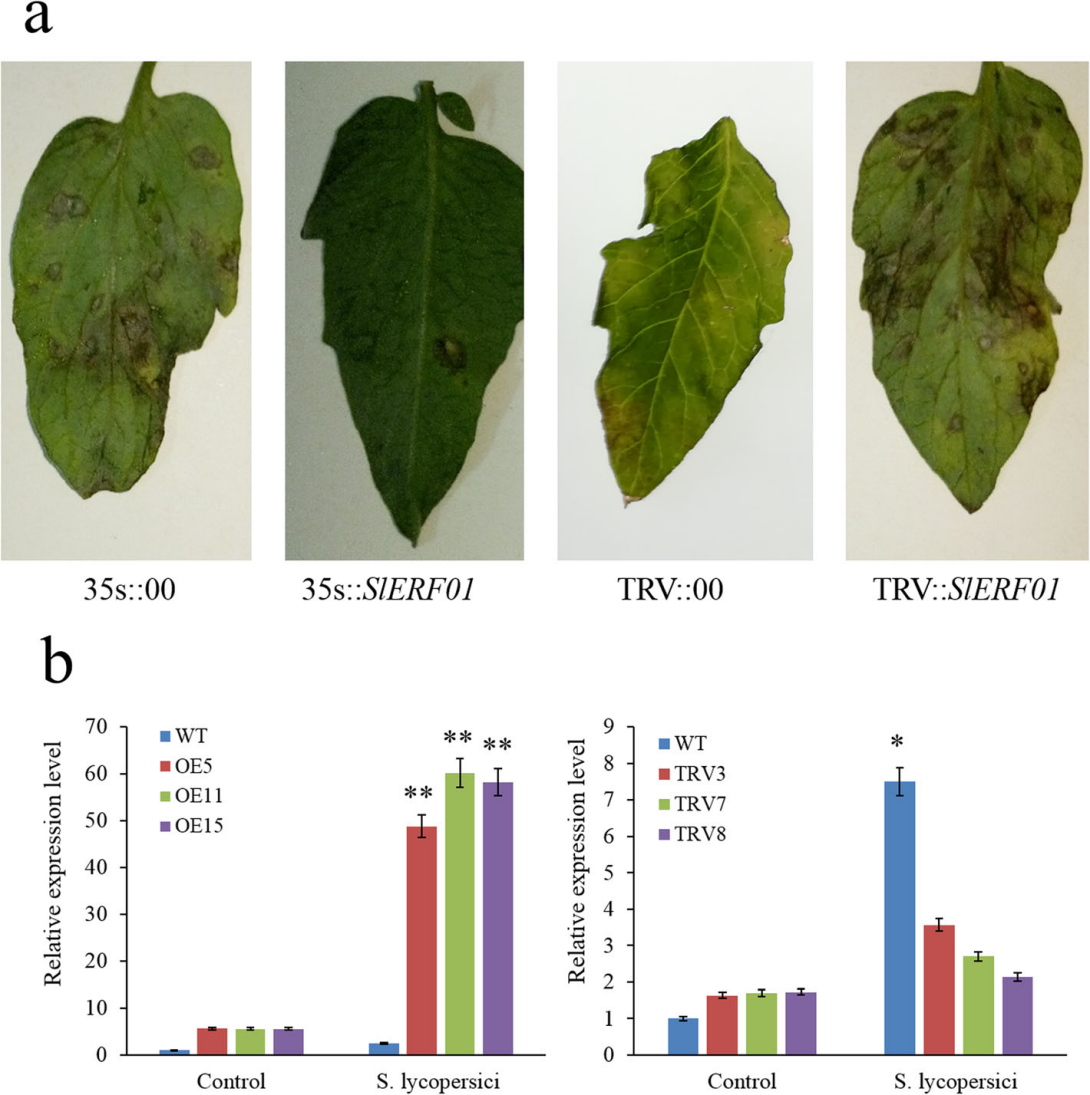
Overexpression of *SlERF01* enhances the disease resistance of tomato. **a** Disease symptoms in wild-type plants, *SlERF01*-overexpressing transgenic plants and silenced plants post inoculation with *S. lycopersici*. The transgenic (35 s::*SlERF01*) plants exhibited a highly resistant phenotype, and the silenced (TRV::*SlERF01*) plants exhibited severe disease symptoms. MT plants transformed with an empty vector (35 s::00); MO resistant plants transformed with a silencing vector (TRV::00). **b** Expression levels of *SlERF01* in wild-type plants, OE plants and VIGS plants. Three OE lines (OE5, OE11 and OE15) and three VIGS (TRV) lines (TRV3, TRV7 and TRV8) were analyzed via qRT-PCR. Three biological replicates were included for each sample. The asterisks indicate significant differences in expression levels between transgenic lines and control lines (**, *P* < 0.01; *, *P* < 0.05, Student’s t-test)

To identify the function of *SlERF01* in tomato resistance to *S. lycopersici*, overexpression and TRV-mediated VIGS vectors were constructed for further analysis. Three *SlERF01-*overexpressing tomato lines presenting the greatest expression (lines 5, 11 and 15) and 3 TRV lines presenting the lowest expression (lines 3, 7 and 8) were ultimately generated for further analysis (Fig. 3). Overexpression of *SlERF01* resulted in a typical HR-type phenotype at 3 dpi with *S. lycopersici*, and the susceptibility symptoms of transgenic SIERF01 overexpression (OE) plants were significantly less severe than those of susceptible plants. Compared with the plants transformed with the empty control vector (35 s::00), the transgenic lines exhibited enhanced resistance to *S. lycopersici* infection*.*

Furthermore, the HR was weaker and slower in *SlERF01*-silenced (TRV) plants than in the plants transformed with the empty control vector (TRV::00). Typical disease lesions were observed on *SlERF01*-silenced plants at 3 dpi, and no obvious susceptible symptoms were observed on the leaves of the TRV::00 plants (Fig. 3a). Furthermore, necrotic lesions and perforated center symptoms were evident on the leaves of the susceptible plants. These results indicated that *SlERF01* promoted tomato resistance to *S. lycopersici*.

**Fig. 4:**
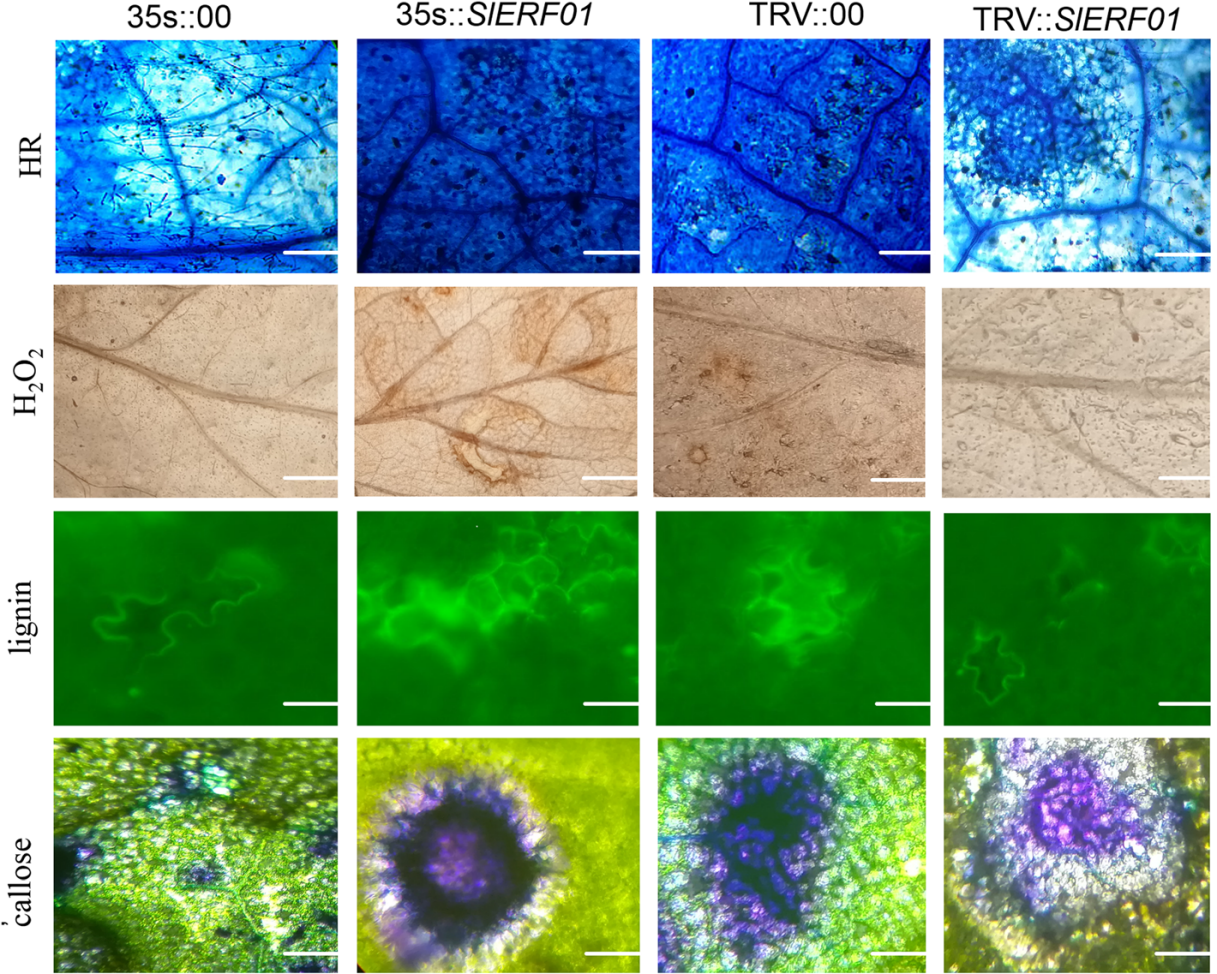
Histopathological observations of HR-related cell death and accumulation of H_2_O_2_, lignin and callose. Similar results were obtained in three independent experiments. Bars = 25 μm

The effects of disease resistance in tomato were also evaluated by examining HR-related cell death and accumulation of H_2_O_2_, lignin, and callose by staining with trypan blue, DAB, TB and AB, respectively (Fig. 4). For trypan blue staining, a strong HR at 3 dpi with *S. lycopersici* was observed in *SlERF01*-overexpressing (35 s::*SlERF01*) plants. In contrast, no visible HR was observed in the empty vector (35 s::00) plants at 3 dpi; the hyphae gradually grew, and the lesions were aggravated and transparent. In contrast to those of the OE plants, the leaves of the *SlERF01*-silenced plants were sensitive to *S. lycopersici* infection. The HR was impaired in the TRV::SlERF01 plants compared with the TRV::00 plants infected with *S. lycopersici* at 3 dpi; hyphal spreading was observed, and the lesions were aggravated and perforated. However, a strong HR was observed on the leaves of the TRV::00 plants. Taken together, these results showed that *SlERF01* can trigger the HR in tomato leaves.

In addition, H_2_O_2_ production was observed in the leaves of the 35 s::*SlERF01* OE tomato plants by DAB staining (Fig. 4). At 3 dpi, compared with that in the TRV::00 plants, the H_2_O_2_ accumulation in the TRV::*SlERF01* plants was too weak to detect. H_2_O_2_ accumulation occurred earlier and stronger in the TRV::00 plants than in the TRV::SlERF01 plants. In contrast, the H_2_O_2_ accumulation occurred earlier and stronger in the OE plants than in the 35 s::00 plants. These results indicated that *SlERF01* can induce H_2_O_2_ production as a defense response to *S. lycopersici* infection. To explore the potential mechanism further, lignin and callose production was analyzed in the 35 s::*SlERF01* OE plants, TRV::*SlERF01* plants and empty vector (35 s::00 and TRV::00) plants at 3 dpi. The accumulation of lignin and callose in the leaves of the 35 s::*SlERF01* OE plants was greater than that in the leaves of the 35 s::00 empty vector plants at 3 dpi (Fig. 4). However, the intensities and areas of fluorescence in the leaves of the TRV::*SlERF01*-silenced plants were weaker than those in the leaves of the TRV::00 plants. On the basis of all of the above results, we conclude that *SlERF01* overexpression enhances the resistance of tomato to *S. lycopersici* compared with that of control plants.

### Silencing of *SlERF01* decreases the expression levels of the defense-related gene *PR1* after infection with *S. lycopersici*

In previous transcriptome sequencing experiments, we found that the expression levels of the differentially expressed genes *SlERF01* and *PR1* were significantly upregulated in the “plant hormone signal transduction” pathway [25]. In the present study, qRT-PCR was used to identify the regulatory relationship between *SlERF01* and *PR* in the “plant hormone signal transduction” pathway. As shown in Fig. 7, once *SlERF01* was silenced, the expression level of *PR1* was significantly suppressed compared with that in the TRV::00 plants. In addition, compared with 35 s::00 plants, the expression levels of the *PR1* gene were significantly upregulated in 35 s::*SlERF01* OE plants (Fig. 7). Therefore, we proposed that *SlERF01* enhances disease resistance to *S. lycopersici* by regulating the expression of the PR1 gene in tomato.

### *SlERF01* may require the SA and JA signaling pathways to enhance disease resistance in tomato

**Fig. 5:**
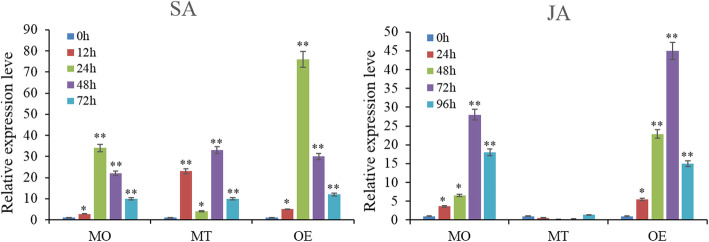
Resistance induced by exogenous SA and JA in response to *S. lycopersici* infection in tomato. MO: resistant cultivar (Motelle), MT: control cultivar (Micro-Tom); OE: overexpression plants. The asterisks indicate significant differences in expression levels between hormone-treated plants and control (water-sprayed) plants. Similar results were obtained in three independent experiments (**, *P* < 0.01; *, *P* < 0.05, Student’s t-test)

The above results show that overexpression of *SlERF01* can improve disease resistance against *S. lycopersici* in tomato. In addition, our previous study showed that *SlERF01* is involved in the significantly enriched Kyoto Encyclopedia of Genes and Genomes (KEGG) pathway “plant hormone signal transduction”. qRT-PCR was used to determine whether the transcript levels of *SlERF01* were associated with SA- and JA-induced resistance in resistant plants during *SlERF01* infection. Compared with the control (water-sprayed) plants, plants treated with 0.2 mM exogenous SA presented approximately 34-fold (in MO resistant plants) and 76-fold (in OE transgenic plants) increases in transcript levels, respectively (Fig. 5). After SA treatment, the expression of *SlERF01* was significantly upregulated and peaked at 24 h; this gene expression pattern was displayed in response to SA induction in both MO resistant plants and OE transgenic plants. In the MT control material, the expression of *SlERF01* was upregulated at 12 h and 48 h after treatment with SA, with a rapid decline at 24 h, exhibiting an irregular change. Therefore, in the MT control material, the expression of *SlERF01* was upregulated at different time points but did not exhibit the same pattern in response to SA induction.

Similarly, treatment with JA also significantly enhanced the expression of *SlERF01*, whose peak expression level was 28-fold (in MO resistant plants) and 45-fold (in OE transgenic plants) greater than that in the control plants. These results showed that *SlERF01* could be significantly upregulated by SA and JA treatment. In the MO resistant material, the expression of *SlERF01* was upregulated in response to JA induction. However, the expression of *SlERF01* was not significantly upregulated at different time points in MT and did not respond to JA induction.

**Fig. 6:**
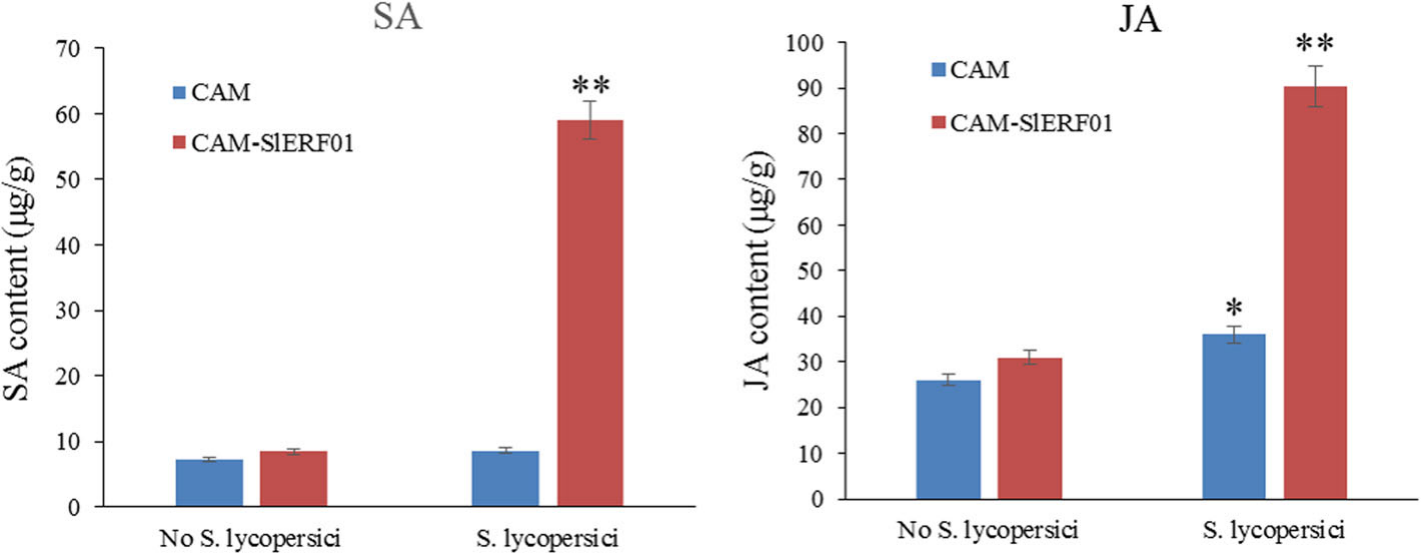
SA and JA hormone levels in *SlERF01*-overexpressing lines. The asterisks indicate significant differences in the expression levels between transgenic lines and controls. The data are from three independent experiments (**, *P* < 0.01; *, *P* < 0.05, Student’s t-test)

It is well known that SA and JA play important roles in the plant defense response to pathogens. To analyze the hormone response to *S. lycopersici* infection, liquid chromatography-mass spectrometry (LC-MS) was performed to measure the JA and SA contents in T1-generation *SlERF01*-overexpressing plants. The SA and JA levels of the T1-generation *SlERF01*-overexpressing tomato plants were significantly greater than those of the control plants after inoculation with *S. lycopersici* (Fig. 6). After inoculation, the SA levels in the *SlERF01*-overexpressing plants were 5-fold greater than those in the empty vector plants, and the JA levels were approximately 3-fold greater than those in the empty vector plants (Fig. 6). Thus, overexpression of *SlERF01* could significantly enhance the production of SA and JA, again indicating that *SlERF01* probably participates in both the SA and JA signaling pathways to improve the disease resistance of tomato to *S. lycopersici*.

**Fig. 7:**
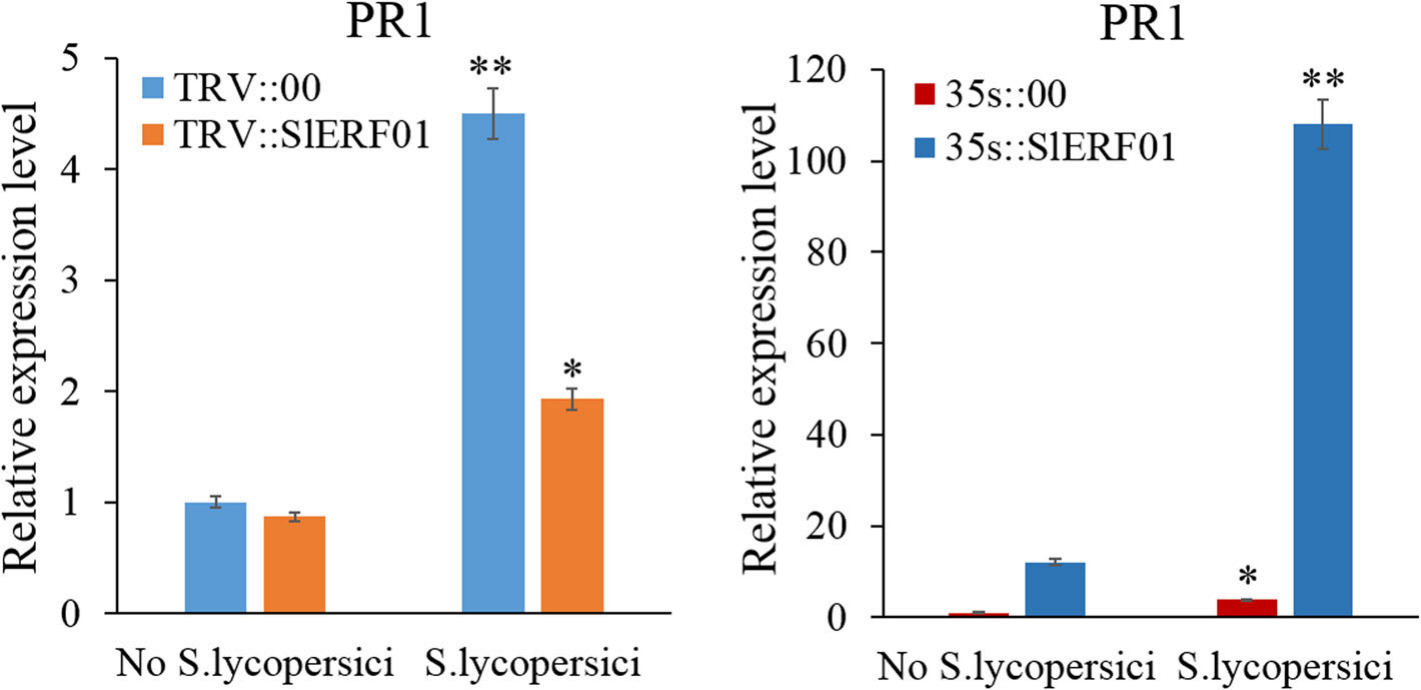
The expression level of the defense-related gene *PR1* in *SlERF01-*silenced and *SlERF01*-overexpressing plants. TRV::00, empty vector plant; TRV::*SlERF01*, *SlERF01-*silenced plant; 35 s::00, plant transformed with an empty vector; 35 s::*SlERF01*, OE plants. The asterisks indicate significant differences in the expression levels between silenced lines and control lines. Similar results were obtained in independent experiments (**, *P* < 0.01; *, *P* < 0.05, Student’s t-test)

**Fig. 8:**
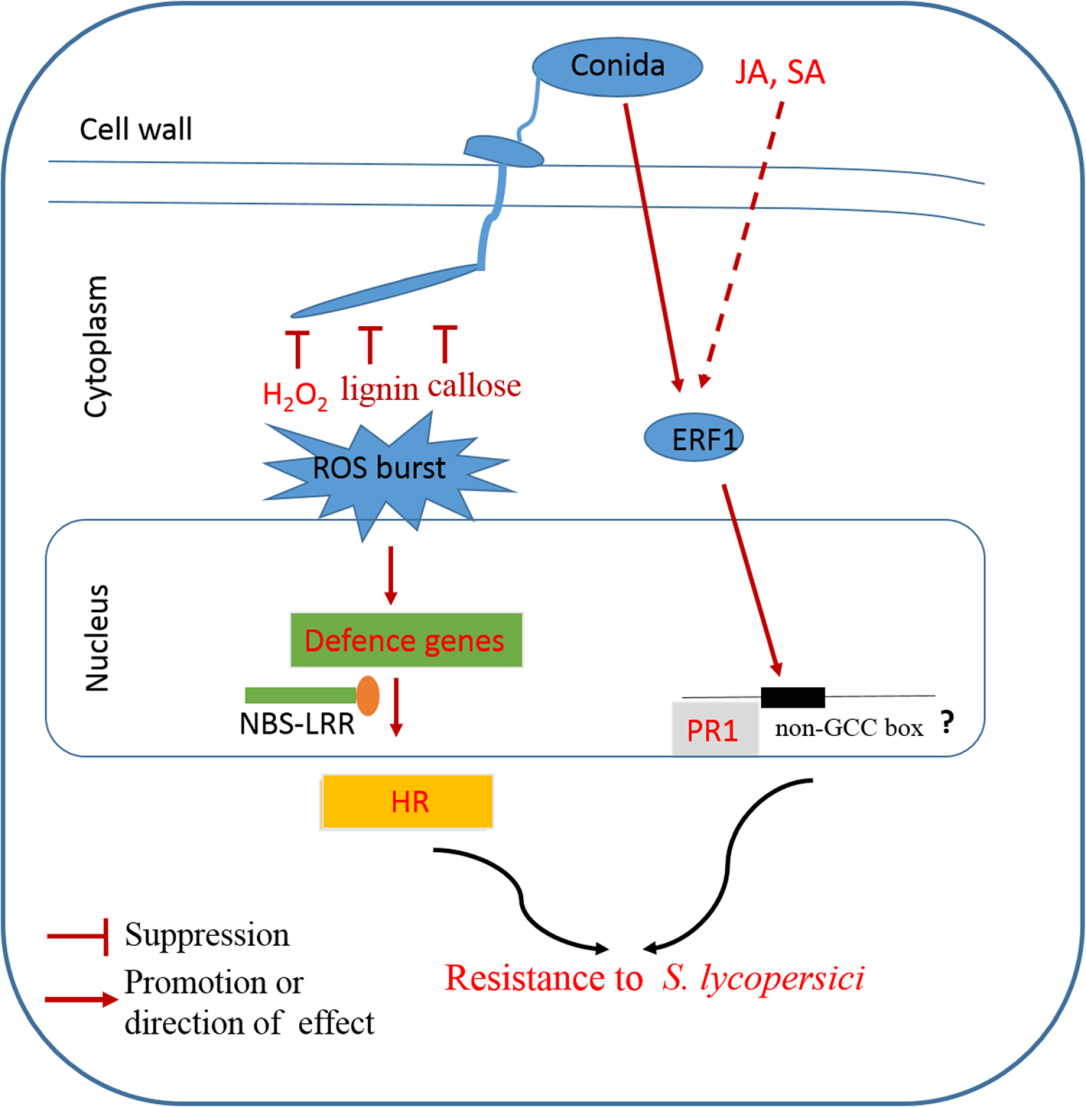
Hypothetical model of the tomato defense response to *S. lycopersici* based on the results of this study

## Discussion

### *SlERF01* is a novel tomato AP2/ERF TF that is localized in the nucleus

To date, approximately 137 genes that encode proteins with conserved AP2/ERF domains have been identified in the tomato genome, and AP2/ERF proteins play an important role in the transcriptional regulation of a variety of abiotic and biotic stress responses. Previous studies have shown that A-subgroup TFs are involved in the regulation of abiotic stress responses. However, nearly all the AP2 genes of the B subgroup have important functions in the biotic stress response. Furthermore, an increasing number of B-subfamily genes have been identified as being involved in resistance to bacterial, fungal and viral diseases [26].

In the present study, *SlERF01* was isolated from tomato, and its expression was shown to be upregulated after *S. lycopersici* treatment. In addition, phylogenetic analysis revealed that *SlERF01* belonged to the B-3 subfamily of ERF proteins, and a few B-3 subfamily members have been shown to regulate plant disease resistance [27]. Analysis of the conserved protein sequences in *SlERF01* revealed a low similarity to ERF1; however, the sequence homology was very high in the ERF domain regions (Fig. 1b). Our results showed that the cDNA of *SlERF01* probably encodes a novel ERF protein that is involved in the disease resistance response. Subcellular localization analysis showed that *SlERF01* is a nuclear-localized protein, which is consistent with the results of previous studies on many ERF proteins.

### *SlERF01* enhances tomato resistance to *S. lycopersici*

It is well known that overexpression of ERFs can enhance plant disease resistance to fungi, bacteria, and viruses. Previous studies have shown that the overexpression of AaERF1 can positively regulate *Artemisia annua* resistance to *Botrytis cinerea* [28]. Furthermore, studies have shown that rice plants expressing the tobacco OPBP1 gene exhibit enhanced resistance to *Magnaporthe grisea* and *Rhizoctonia solani* [29].

The results of our present study showed that overexpression of *SlERF01* could significantly enhance resistance to *S. lycopersici* infection compared with that of control plants*.* Typical disease lesions were observed on *SlERF01*-silenced plants, with no obvious susceptibility symptoms observed on TRV::00 plants. Moreover, studies have indicated that the HR and the accumulation of H_2_O_2_, lignin and callose are stronger in resistant cultivars than in susceptible cultivars, leading to improved disease resistance [30, 31]. Consistent with these previous studies, our study showed that overexpression of *SlERF01* not only led to HR-induced cell death but also increased the accumulation of H_2_O_2_, lignin and callose in transgenic tomato plants compared with control plants. These results indicated that *SlERF01* may also participate in resistance against *S. lycopersici* via ROS signaling (Fig. 8).

### *SlERF01* positively regulates the expression of *PR1* and enhances tomato disease resistance

Some ERF TFs, such as OsERF1, Pti4 and AtERF1, were recently suggested to play a role in the disease resistance response. As discussed in the introduction, overexpression of ERFs in plants can enhance plant disease resistance by regulating *PR* gene expression [32]. The regulation of *PR* gene expression by ERF TFs by binding to GCC boxes or to DRE/CRT cis-acting elements within gene promoter regions has been extensively studied [33–35]. Furthermore, studies have shown that sequences flanking GCC boxes affect binding efficiency, suggesting that multiple ERFs probably regulate various gene sets [36]. Therefore, ERFs may directly or indirectly regulate PR gene expression and enhance plant resistance to disease. Here, we also showed that overexpression of the *SlERF01* gene upregulated the expression of the *PR1* gene and enhanced the tomato resistance to *S. lycopersici.*

### *SlERF01* may require the SA and JA signaling pathways to enhance disease resistance in tomato

In previous transcriptome sequencing experiments, we found that *SlERF01* expression was induced by *S. lycopersici* in both resistant and susceptible materials and was highly upregulated in the resistant material after inoculation with *S. lycopersici* [25]. Furthermore, SA and JA are important signaling molecules that are involved in the disease resistance response to biotic and abiotic stress [37, 38]. Our results showed that the expression of *SlERF01* could be induced by exogenous SA in MO resistant plants and OE transgenic plants, suggesting that *SlERF01* is probably the responsive component of the SA signaling pathways. Previous studies have also shown that exogenous application of SA can induce the expression of *PR* genes and enhance resistance to multiple pathogens [39]. Our data were consistent with previous findings in which ERF1 was responsive to ET and SA through activated expression of downstream *PR* genes [19]. However, the expression of *SlERF01* exhibited an irregular pattern and was downregulated in MT susceptible plants at 24 h after SA treatment, indicating that *SlERF01* presented distinct expression characteristics between resistant plants and susceptible plants. *SlERF01* may be involved in crosstalk in response to pathogen attack via synergistic interactions of various signaling pathways. These results were consistent with the regulation of *AhRRS5* differing between resistant and susceptible peanut varieties [40]. In addition, the SA and JA/(ET) signaling pathways were identified as being antagonistic or synergistic in the disease resistance response [41–43]. Previous studies have shown that OsERF1 integrates the SA and JA signaling pathways in the defense response against pathogens [44]. Our results consistently showed that *SlERF01* was also induced by exogenous JA, suggesting that *SlERF01* probably plays a role in mediating communication between the SA and JA signaling pathways. Previous studies have shown that the ROS and SA pathways have parallel functions to ensure optimal induction of SAR [45]. Combined with the results of the above studies, our results showed that *SlERF01* not only responded to SA and JA but also increased the accumulation of H_2_O_2_, lignin and callose in transgenic tomato plants. Here, we propose that *SlERF01* plays a critical role in the crosstalk among SA, JA and ROS, providing resistance to *S. lycopersici* invasion (Fig. 8).

## Conclusions

In this study, we identified *SlERF01* as a novel gene in tomato encoding an AP2/ERF TF that localizes to the nucleus. Analyses of overexpression and gene silencing data revealed that *SlERF01* positively regulates tomato resistance to *S. lycopersici*. Interestingly, *SlERF01* plays a key role in multiple SA, JA and ROS signaling pathways to provide resistance to invasion by *S. lycopersici.* Preliminary functional analysis demonstrated that *SlERF01* induces disease resistance by upregulating the expression of the PR1 gene. This study ultimately provides valuable resources for future studies of the molecular mechanisms involved in disease resistance and breeding strategies for tomato varieties.

## Methods

### Plant materials and *S. lycopersici* inoculation

Tomato plants of the resistant cultivar Motelle (MO) were provided by the Chinese Academy of Agricultural Sciences. Seedlings of the transgenic line Micro-Tom (MT) and *Nicotiana benthamiana* were obtained from our laboratory. Tomato and tobacco plants were subsequently grown in a greenhouse at 25–28 °C and 60% relative humidity under a 14 h/10 h light/dark photoperiod.

*S. lycopersici* was isolated from tomato plants and plated on potato dextrose agar (PDA) in Petri dishes at 25–28 °C for 10 days under a 12 h/12 h photoperiod. Afterward, 4-week-old tomato seedlings of MO, Moneymaker and MT were inoculated with a conidial suspension (1 × 10^4^ conidia mL^− 1^), while control plants were sprayed with sterilized water. The plants were maintained in a greenhouse (25–28 °C) under a relative humidity of > 80%. The disease indexes were evaluated post inoculation, and leaves were harvested at 0 and 3 days post inoculation (dpi) for further analysis.

### Gene cloning and bioinformatic analysis

The 5′- and 3′-ends of cDNA sequences were cloned by homologous recombination via PCR Cloning Kit. Specific primers used for the target sequence were designed via Primer 6.0 software, and the target gene *SlERF01* was cloned via PCR implemented in accordance with the following reaction protocol: 94 °C for 3 min; 35 cycles of 94 °C for 30 s, 60 °C for 45 s, and 72 °C for 30 s kb^− 1^; and 72 °C for 10 min. A part-CAM-SLERF01 vector was constructed for the identification of positive clones. All the primers used in the study are shown in Table S1.

The *SlERF01* sequence was examined by checking the NCBI Conserved Domain Database (CDD) (https://www.ncbi.nlm.nih.gov/Structure/cdd/wrpsb.cgi), and the identified sequences were analyzed via DNAMAN 5.0 (Data S2). A phylogenetic tree of the AP2/ERF family proteins of tomato was subsequently constructed by MEGA 5.2.

### Subcellular localization

The full-length *SLERF01* open reading frame (ORF) without the termination codon was amplified via PCR in conjunction with a high-fidelity polymerase together with the primers GFP-*SLERF01*-F and GFP-*SLERF01*-R. A pCAM35::*SlERF01*-GFP fusion construct was prepared by inserting the PCR products into a pCAM35::GFP vector between its KpnI and XbaI sites. The pCAM35::GFP (control) and pCAM35::*SLERF01*-GFP vectors were subsequently transformed into *Agrobacterium tumefaciens* GV3101. Single clones were selected and then cultured in Luria-Bertani (LB) liquid media containing corresponding antibiotics. The transformed *Agrobacterium* cells were concentrated by centrifugation, after which they were harvested, diluted to an OD_600_ of 0.4, and injected into *N. benthamiana* leaves via a syringe. Two days after agroinfiltration, the green fluorescent proteins (GFPs) were imaged by a laser scanning confocal microscope (FV10-ASW, Olympus).

### Transformation of tomato

The full-length coding DNA sequence (CDS) of *SlERF01* was amplified via PCR and cloned into a part-CAM vector harboring XhoI and XbaI sites. A pCAM-*SLERF01* overexpression vector was constructed, and the pCAM-*SlERF01* recombinant plasmid and the pCAM plasmid were transferred into *A. tumefaciens* strain GV3101 (BioVector NTCC Inc., Beijing, China). The pCAM-*SlERF01* (overexpression) and pCAM (empty) vectors were transferred into the susceptible cultivar MT via a tomato genetic transformation technique [46]. Ten-day-old tomato seedlings were used as explants and precultured for 2 days on MR (Murashige and Skoog (MS) media supplemented with 0.2 mg l^− 1^ zeatin and 1.0 mg l^− 1^ indoleacetic acid (IAA), pH 5.8) media.

A single colony of *A. tumefaciens* was selected from LB liquid media that was supplemented with corresponding antibiotics. Bacterial cells were then collected, after which tomato cotyledons were immersed in the bacterial suspension for 3–5 min and cocultivated for 2 days. Infected cotyledons were transferred to suitable media and allowed to grow for 2 weeks, and the explants were subcultured every 3 weeks. After acclimatization, plantlets with well-developed roots were transplanted into soil.

Two different *A. tumefaciens* strains were used for virus-induced gene silencing (VIGS). One carried TRV1, which encoded viral proteins needed for replication and movement, while the other, TRV2, harbored the coat protein and sequence used for VIGS [47]. The target sequence of *SlERF01* was amplified via PCR with specific primers. After digestion with EcoRI and BamHI, the TRV vector was ligated to the PCR product. TRV::*SlERF01*, TRV::00 and TRV::*PDS* vectors were constructed and propagated in LB media that containing 50 mg mL^− 1^ kanamycin. The recombinant plasmids were then transferred into *A. tumefaciens* strain GV3101, after which the transformed cells were cultured in induction media (10 mM 2-(N-morpholino) ethanesulfonic acid (MES), 10 mM MgCl_2_, 2.50 μg mL^− 1^ kanamycin, 100 μg mL^− 1^ rifampicin and 200 μM acetosyringone) to an OD_600_ of 0.3. Lst, TRV1 and TRV2 were mixed together at a volumetric ratio of 1:1 and incubated for 3 h; MO plants at the 3–4-leaf stage were then infiltrated with each mixture via a 1 mL syringe containing approximately 0.5–1 mL of the *Agrobacterium* cell culture solution. The treated plants were sampled at indicated time points for further analysis, and 3 biological replicates were included in the test.

### Real-time quantitative reverse transcription-polymerase chain reaction (qRT-PCR) analysis and determination of physiological indexes

Expression analysis of the overexpression and VIGS plants was performed via qRT-PCR. Total RNA was extracted from tomato leaves by TRIzol reagent [48]. cDNA was synthesized by a reverse transcription kit (TaKaRa) according to the manufacturer’s instructions. The qRT-PCR system consisted of 10 μL of 2× TransStart Top Green qPCR SuperMix (TransGen, China), 0.5 μL of forward/reverse primers, and 2 μL of cDNA template, and ddH_2_O was added to bring the total volume to 20 μL. The qRT-PCR program was as follows: 95 °C for 10 min, followed by 40 cycles of 95 °C for 5 s, 62 °C for 15 s and 72 °C for 30 s. The 2^–∆∆CT^ method [49] was subsequently used to analyze the qRT-PCR data, with *EF1α* serving as a reference gene [50]. The qRT-PCR primers used are listed in Table S1.

For exogenous hormone treatment, 0.2 mM SA and 0.4 mM JA solutions were sprayed onto tomato plants (the control plants were sprayed with water) at different time points (SA: 0, 12, 24, 48 and 72 h; JA: 0, 24, 48, 72 and 96 h). The levels of the endogenous SA and JA hormones were measured via high-performance liquid chromatography (HPLC). SA and JA were extracted from the leaves according to a modified method described by Llugany et al. [51], after which their concentrations were measured by an AB SCIEX QTRAP 5500 instrument (USA) according to the manufacturer’s instructions. Samples were collected from three individual plants for analyses of the SA content, JA content and gene expression. Data from three independent experiments were statistically analyzed according to Student’s t-tests, and *P* < 0.05 was considered statistically significant.

### Microscopy observations

Trypan blue staining [52], 3,3-diaminobenzidine (DAB) staining, toluidine blue (TB) staining and aniline blue (AB) staining were used to observe the progression of *S. lycopersici* infection and the production of H_2_O_2_, lignin and callose in *SlERF01*-overexpressing and *SlERF01*-VIGS plants. The leaves were collected at 0 and 3 days after inoculation.

Cell death was observed by the use of TB staining, with destaining in Farmer’s solution (95% ethanol:chloroform:acetic acid at a volumetric ratio of 6:3:1) for 3 h and boiling in 0.1% trypan blue solution at 65 °C for 2 h, followed by transfer to a saturated chloral hydrate solution for 4 h. The leaves were ultimately observed under a light microscope.

The production of H_2_O_2_ was detected via DAB staining [53]. Infected tomato leaves were incubated in 0.1% DAB solution at room temperature in the dark for 12 h and then boiled in a 96% ethanol solution for 10 min. The leaves were ultimately observed under a light microscope. Lignin was observed by the use of the TB staining method [54]. The infected tomato leaves were placed in formaldehyde:acetic acid:ethanol (FAA) solution for 24 h and then stained with a 0.05% TB solution. The leaves were subsequently observed under a light microscope. Callose was detected by the use of the AB staining method [55]. The infected tomato leaves were placed in FAA solution, cleared with 100% ethanol solution and then stained with 0.07 M K_2_HPO_4_ in a 0.01% AB solution for 24 h. The leaves were ultimately observed under a fluorescence microscope. Leaf samples were collected from three individual plants for analyses of the HR, H_2_O_2_ production, and lignin and callose accumulation.

## Supplementary information

**Additional file 1: Table S1.** Primers used in this study.

**Additional file 2: Table S2.**

**Additional file 3: Table S3.**

## Data Availability

The datasets supporting the results of this study are included with the article and its additional files (Table S2 and Table S3). The materials are available upon request by contacting the corresponding author. The data concerning the phylogenetic tree and sequence alignment of *SlERF01* are shown in Fig. 1. The data concerning the subcellular localization of *SlERF01* are shown in Fig. 2. The data concerning the overexpression of *SlERF01* in tomato are shown in Fig. 3. The data concerning the histopathological observations of HR-related cell death and accumulation of H_2_O_2_, lignin and callose are shown in Fig. 4. The data concerning the resistance induced by exogenous SA and JA against *S. lycopersici* infection in tomato are shown in Fig. 5. The data concerning the hormone level analysis of the control and transgenic lines are shown in Fig. 6. The data concerning the expression levels of *SlERF01* and PR1 are shown in Fig. 7. The data concerning the hypothetical model of the tomato defense response to *S. lycopersici* are shown in Fig. 8.

## Abbreviations

*S. lycopersici*: *Stemphylium lycopersici*
PTI: PAMP-triggered immunity
ETI: Effector-triggered immunity
ROS: Reactive oxygen species
HR: Hypersensitive response
SAR: Systemic acquired resistance
PR1: Pathogenesis-related protein 1-like
R gene: Resistance gene
qRT-PCR: Real-time quantitative reverse transcription-polymerase chain reaction
VIGS: Virus-induced gene silencing
SA: Salicylic acid
JA: Jasmonic acid
