# Supplementary material for: Functional analysis of the SlERF01 gene in disease resistance to S. lycopersici
Source: BMC Plant Biol. 2020 Aug 15;20:376. doi: 10.1186/s12870-020-02588-w (PMC7429758; doi:10.1186/s12870-020-02588-w)
Supplement: Supplementary file 1 — Additional file 1: Table S1. Primers used in this study. [file 12870_2020_2588_MOESM1_ESM.docx]

Table S1.Primers used in the text.

| Primer name | Forward primer sequence (5’-3’) | | Reverse primer sequence (5’-3’) | |
| --- | --- | --- | --- | --- |
| V-*PR1* | | CGAATTCATCTGAAAAGGCTTCCCCCG | | CGGATCCACACAAAAGAAGCCCAACGC |
| V-*SLERF01* | | CGAATTCTCCGAAACAGTCACATCGCA | | CGGATCCAGCATCTTCCGCGCTATCAA |
| *PR1-*F | | ATCTGAAAAGGCTTCCCCCG | | ACACAAAAGAAGCCCAACGC |
| *SlERF01-*F | | TCCGAAACAGTCACATCGCA | | AGCATCTTCCGCGCTATCAA |
| *EFa1* | | CCACCAATCTTGTACACATCC | | AGACCACCAAGTACTACTGCAC |
